# Older Student Experiences: A Critical Examination of Aging on Campus

**DOI:** 10.1093/geroni/igab046.2015

**Published:** 2021-12-17

**Authors:** Cassandra Barragan, Sarah Walsh

**Affiliations:** 1 Eastern Michigan University, Ypsilanti, Michigan, United States; 2 Eastern Michigan University, Eastern Michigan University, Michigan, United States

## Abstract

There is overwhelming evidence that the number of older learners on college campuses has been steadily increasing since the 1970s. The needs of older learners differ from traditional students, and many services and resources available at higher education institutions are geared towards students aged 18-25 (Silverstein, Choi, & Bulot, 2001). Age Friendly University (AFU) principles highlight the need to consider older learners at a university and provide structure to evaluate programs and practices and to enhance inclusion and diversity based upon age. This study examined how an AFU designated university is working to better understand their older students. Methods: A web-based pilot survey of older learners (N=248) asked all students ages 40 and older a series of questions regarding motivation to attend school, barriers and supports, campus environment, and connection with AFU principles. Analysis: A regression analysis found that older learners who felt more welcomed by faculty (p=.001), administration (p=.002),and student organizations (p=.026) were more likely to feel connected to campus, and younger-older students (p=.031) and those who did not feel their job was a barrier to attending school (p=.037) were more likely to feel satisfied with their level of engagement on campus. Additionally, older learners felt the AFU principles were demonstrated by their university. Discussion: The experiences of older learners are important as we continue to see higher numbers of students over the age of 40. Our results demonstrate the need to engage older learners as part of diversity and inclusion efforts to facilitate connection to the campus community.

